# Ingredients in Zijuan Pu’er Tea Extract Alleviate β-Amyloid Peptide Toxicity in a *Caenorhabditis elegans* Model of Alzheimer’s Disease Likely through DAF-16

**DOI:** 10.3390/molecules24040729

**Published:** 2019-02-18

**Authors:** Fangzhou Du, Lin Zhou, Yan Jiao, Shuju Bai, Lu Wang, Junfeng Ma, Xueqi Fu

**Affiliations:** 1 National Engineering Laboratory for AIDS Vaccine, School of Life Sciences, Jilin University, Changchun 130012, China; du_fangzhou@163.com (F.D.); 18744021315@163.com (L.Z.); jiaoyan@novogene.com (Y.J.); baisj16@mails.jlu.edu.cn (S.B.); luwang_0925@163.com (L.W.); 2 Key Laboratory for Molecular Enzymology and Engineering, The Ministry of Education, School of Life Sciences, Jilin University, Changchun 130012, China

**Keywords:** Alzheimer’s disease, oxidative stress, *C. elegans*, amyloid-β

## Abstract

Amyloid-β, one of the hallmarks of Alzheimer’s disease (AD), is toxic to neurons and can also cause brain cell death. Oxidative stress is known to play an important role in AD, and there is strong evidence that oxidative stress is associated with amyloid-β. In the present study we report the protective effect of Zijuan Pu’er tea water extract (ZTWE) and the mixture of main ingredients (+)-catechins, caffeine and procyanidin (MCCP) in ZTWE on β-amyloid-induced toxicity in transgenic *Caenorhabditis elegans* (*C. elegans*) CL4176 expressing the human Aβ_1–42_ gene. ZTWE, (+)-catechins, caffeine, procyanidin and MCCP delayed the β-amyloid-induced paralysis to different degrees. The MCCP treatment did not affect the transcript abundance of amyloid-β transgene (*amy-1*); however, Thioflavin T staining showed a significant decrease in Aβ accumulation compared to untreated worms. Further research using transgenic worms found that MCCP promoted the translocation of DAF-16 from cytoplasm to nucleus and increased the expression of superoxide dismutase 3 (SOD-3). In addition, MCCP decreased the reactive oxygen species (ROS) content and increased the SOD activity in CL4176 worms. In conclusion, the results suggested that MCCP had a significant protective effect on β-amyloid-induced toxicity in *C. elegans* by reducing β-amyloid aggregation and inducing DAF-16 nuclear translocation that could activate the downstream signal pathway and enhance resistance to oxidative stress.

## 1. Introduction

Alzheimer’s disease (AD) is a neurodegenerative disease characterized by cerebral degeneration, neuronal cell death, tau tangles in the affected brain nerve cells [1] and the accumulation of 39–43 amyloid-β (Aβ) in plaques [2]. Abnormal accumulation of amyloid-β (Aβ) in the brain is considered important in this disease. Aβ-aggregation has also been linked to increased oxidative stress causing neuronal injury and death. It is believed that preventing deposition of Aβ oligomers, reduction of oxidative stresses, or activation of disease modifying pathways could reduce the onset of AD [3,4]. Therefore, many efforts have been made to develop strategies targeting Aβ for the prevention and treatment of AD [5].

For its unique color, flavor, and taste, tea is consumed worldwide as one of the most popular aromatic beverages. Green tea, Oolong tea and black tea, they are all produced from the plant *Camellia sinensis*. Tea includes many ingredients, particularly polyphenolic compounds existed in tea, which can reduce the risk of multiple diseases. In addition, catechinic acid, a kind of flavonoid abundant in tea, is known to be one of the most important components of tea, especially green tea, showing its health contributing potential [6,7]. It also shows its health contribution potential in retinal degeneration [8], cardiotoxicity [9] and Alzheimer’s disease [7,10].

Zijuan tea plant (*Camellia sinensis var. kitamura*) is a cultivar developed from an individual plant of *C. sinensis var. kitamura* obtained from Yunnan Province in China. It has purple stems, buds, and leaves, a light purple calyx and pedicle, and pale purple fruit skin. Its leaves are processed into Zijuan sun-dried green tea by multiple procedures, including fixation, rolling, and sun drying. Zijuan green tea can be further manufactured into the more valuable Zijuan Pu’er tea by appropriate wetting and solid-state fermentation [11]. Fermented Zijuan Pu’er tea is brown. Its liquor appears thick and brightly red with a rich taste without bitterness, and it has a unique fragrance. Several Chinese researchers have reported that, due to its ‘large molecular pigments’ content, fermented Zijuan tea had effects on lowering blood lipids in rats consuming a high-lipid diet [11]. Zijuan Pu’er tea is also known that its ethanol-soluble pigment has anti-oxidative activities [12]. Furthermore, some researchers isolated the compound of anthocyanin from Zijuan tea and well evaluated its antioxidant activities [13]. 

*Caenorhabditis elegans* is a highly suitable animal model for studying the effects of bioactive components which might have relativity to human health. This is partly due to its highly conservation to the human genome [14]. Many stress-induced pathways and their components studied in *C. elegans* are similar to those of humans. In addition, *C. elegans* has a short lifespan and can easily be cultured in the laboratory. These characteristics make the *C. elegans* a suitable model for studying the biochemical and molecular responses to all kinds of environmental stresses. *Caenorhabditis elegans* has been recently used as a model to study the mechanism of β-amyloid (Aβ) toxicity and test the activity of potential anti-AD agents [15,16,17]. Transgenic *C. elegans* strains that express human amyloid-β (Aβ) accelerate further understanding of the mechanisms of Aβ-toxicity in biological systems and can be used for in vivo drug screening [16,17]. Irene Sola using transgenic CL4176 and CL2006 *C. elegans* detected the anti-Alzheimer’s disease effects of synthetic compounds [18]. Natural products such as extracts from ginkgo leaves [19,20], *Glycyrrhiza uralensis* [20], cranberry [21], camu-camu [22], Royal jelly [23] and others were found to reduce Aβ-induced paralysis in *C. elegans*. Furthermore, usage of this model also revealed the molecular mechanisms of these products’ activities.

In the present study, we employed CL4176, a transgenic *C. elegans* expressing Aβ in body wall muscle cells, to explore the beneficial effects of ZTWE in AD prevention. ZTWE and the mixture of the three main ingredients in ZTWE—(+)-catechins, caffeine, procyanidins (MCCP)—both significantly delayed the progression of body paralysis. Further study showed that the pretreatment with MCCP was also effective. These findings indicated that MCCP played a protective role in response to Aβ toxicity. Genetic analyses suggested that this beneficial effect might be dependent on the DAF-16.

## 2. Results

### 2.1. ZTWE Delays the Progression of Paralysis in AD Worms

**Figure 1:**
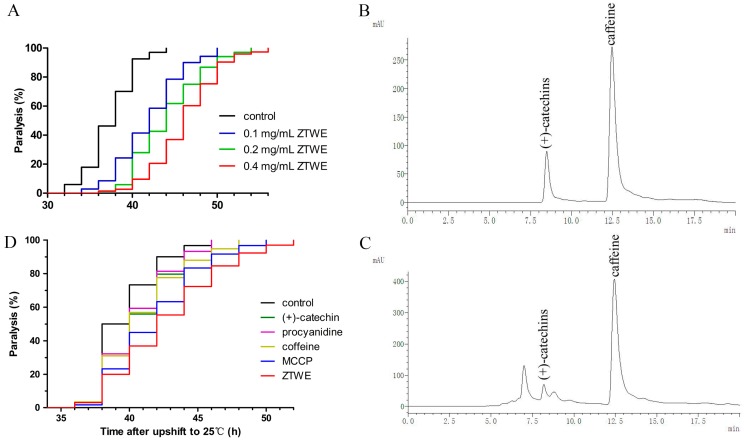
ZTWE and MCCP alleviated Aβ-induced paralysis in transgenic *C. elegans* strain CL4176. (**A**) The ZTWE-treated CL4176 worms showed delayed progression of body paralysis as compared with control worms. Mean ± SD for control, 0.1, 0.2 and 0.4 mg/mL were 37.37 ± 2.70, 41.80 ± 2.17, 42.54 ± 3.85 and 46.19 ± 3.99 respectively. The paralysis assay was repeated at least three times (control with 0.1, 0.2 and 0.4 mg/mL ZTWE, *p* < 0.001). The HPLC chromatogram of (+)-catechins and caffeine standards (**B**) and 2 mg/mL ZTWE (**C**). (**D**) Incorporation of pure (+)-catechins, caffeine, procyanidins and MCCP into agar media dishes also slow induced paralysis, mean ± SD for control, (+)-catechins, caffeine, procyanidins and MCCP were 39.73 ± 2.31, 40.71 ± 2.62, 41.00 ± 2.98, 41.93 ± 3.29 and 42.77 ± 3.95 respectively (control with (+)-catechin, caffeine and procyanidins, *p* < 0.05; control with MCCP and ZTWE, *p* < 0.001).

In consideration of the potential influence of ZTWE for treating AD, we examined whether supplementation of ZTWE in AD worms may affect the progression of paralysis induced by Aβ toxicity. We recorded the time required for worms from up-shifting temperature to appearing paralysis and calculated *p*-value by counting and comparing the time of all worms in different groups. As compared to the control, the onset of Aβ-induced paralysis was dramatically delayed with ZTWE treatment at concentrations of 0.1–0.4 mg/mL, and the effect was concentration dependent (Figure 1A). Considering that the paralysis of CL4176 is driven by Aβ toxicity, these observations suggested that ZTWE alleviated AD symptoms by protecting against Aβ-induced toxicity in worms.

The main components of ZTWE are catechins and caffeine, and ZTWE contains more procyanidins than other teas. To investigate whether the effect of reduced paralysis from ZTWE might be due to (+)-catechins, caffeine or procyanidins, we tested the content of (+)-catechins, caffeine and procyanidins in ZTWE by HPLC and cyanide methods. Figure 1B showed the chromatogram of (+)-catechins and caffeine standards. We draw standard curves according to different peak areas corresponding to different standard substance concentrations ((+)-catechins and caffeine) and according to the absorbance of different standard substance concentrations (procyanidins) (Table 1), and then used these standard curves to calculate the concentration of (+)-catechins, caffeine and procyanidins in 20 mg/mL ZTWE. The results were 0.8265, 1.558 and 0.1324 mg/mL, respectively. We assayed the effect of (+)-catechins, caffeine and procyanidins at the concentration equivalent to that found in 20 mg/mL ZTWE. Addition of pure (+)-catechins, caffeine and procyanidins also had significant effects and the mixture of catechins, caffeine and procyanidins (MCCP) had a more significant effect which was a little weaker than the effect of 0.4 mg/mL ZTWE (Figure 1D). The results indicated that MCCP played an important role in ZTWE. 

### 2.2. MCCP Reduces Aβ Deposits

**Figure 2:**
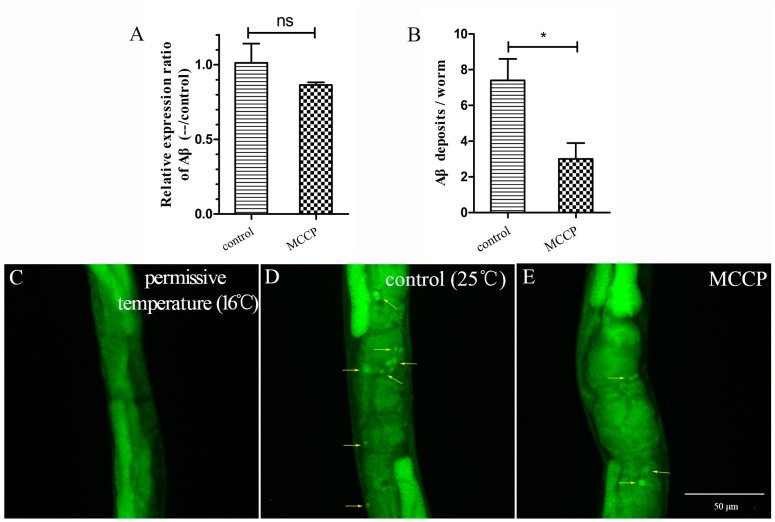
Effect of MCCP treatment on Aβ transgenic expression and Aβ accumulation. (**A**) Quantitative real-time PCR measured Aβ mRNA levels of CL4176 transgenic worms at 36 h after temperature shifted to 25 °C, average of three biologically independent experiments (ns meant that the difference was not significant). (**B**) Statistics on the number of Aβ deposits in different groups of worms, and each group had 10 worms, error bar represented the mean ± SEM (* *p* < 0.05). (**C**–**E**) Thioflavin T staining of transgenic *C. elegans*. unc-54/Aβ_1–42_ transgenic *C. elegans* (CL4176) stained with thioflavin T. Note: muscle thioflavin T-relative deposits. Arrows indicate the Aβ aggregation deposits.

As MCCP played an important role in ZTWE, next we determined whether MCCP might alleviate Aβ-induced paralysis by reducing expression of the Aβ transgene. Quantitative real-time PCR was used to quantify Aβ mRNA levels. Both MCCP-treated and control worms were harvested 36 h after the temperature shifted to 25 °C. As shown in Figure 2A, Aβ transcript level did not show any significant differences. Since MCCP did not affect the expression of Aβ mRNA level, we then performed the thioflavin T staining assay to determine whether the delayed paralysis in transgenic *C. elegans* CL4176 was due to the reduction in the amount of Aβ_1–42_ oligomer. We observed that compared with worms at permissive temperature (16 °C), a large amount of Aβ deposits appeared in worms after temperature shifted to 25 °C (Figure 2C,D). This phenomenon verified the explanation of this worm strain. As shown in Figure 2D,E, worms treated with MCCP had less thioflavin T-positive Aβ deposits than control worms. Figure 2B showed the statistics of Aβ deposits in each group and indicated that MCCP treatment significantly reduced the Aβ deposits in CL4176 worms.

### 2.3. MCCP Might Requires DAF-16 to Protect AD Worms against Aβ Toxicity

**Figure 3:**
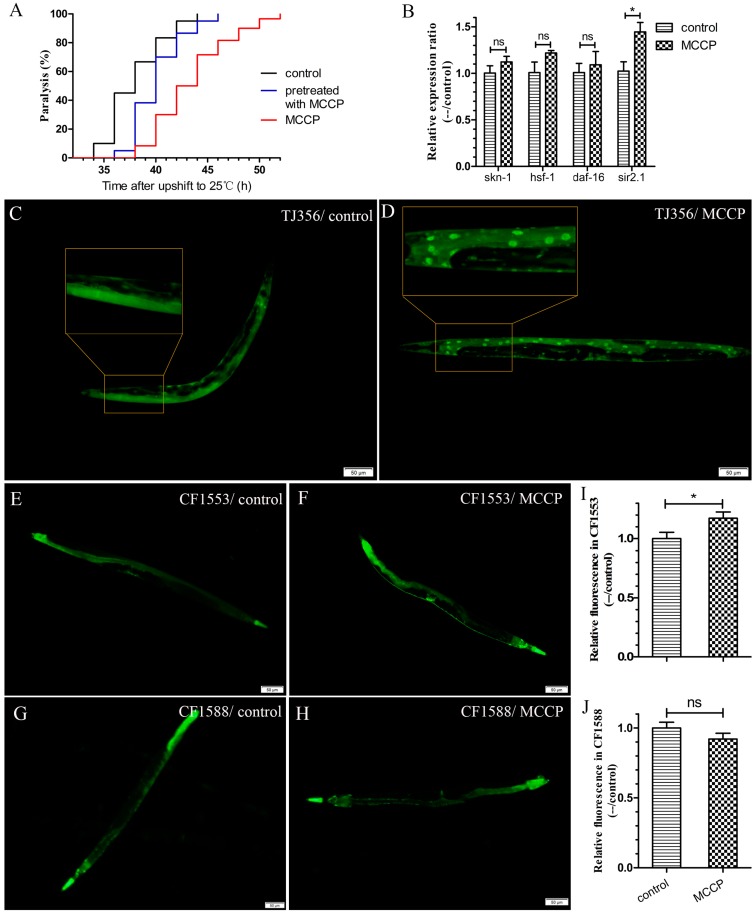
MCCP exposure upregulated the *sir-2.1* transcript level and promoted the DAF-16 nuclear translocation. (**A**) CL4176 worms were incubated at 16 °C for 48 h on agar media dishes containing MCCP, either moved to control dishes or maintained on MCCP dishes, and then induced to express Aβ_1–42_. It was indicated that obvious protection against paralysis was observed even in worms shifted to control dishes before induction of Aβ_1–42_ expression. Mean ± SD for control, pretreatment with MCCP and MCCP were 38.00 ± 2.73, 40.10 ± 2.45 and 43.43 ± 3.67 respectively (control with pretreatment with MCCP and MCCP, *p* < 0.001). (**B**) The transcript levels of *sir-2.1*, *hsf-1*, *daf-16*, and *skn-1* in CL4176 worms treated with and without MCCP were quantified using qRT-PCR. The data was obtained from three independent experiments. (**C**,**D**) MCCP treatment was able to accelerate nuclear localization of DAF-16::GFP in *C. elegans* (TJ356). Data was obtained from three independent experiments (10 worms each). (**E**,**H**) The expression of SOD-3 in SOD-3::GFP worms (CF1553 and CF1588) treated with or without MCCP. Data was obtained from three independent experiments (10 worms each). (**I**,**J**) The fluorescence intensity from SOD-3::GFP in day-4-adults was calculated by Image pro plus. Comparisons between treatments and controls were significant, * *p* < 0.05. Data were obtained from three independent experiments (10 worms in each group). (ns meant that the difference was not significant).

In order to determine whether MCCP was needed to be present in the media to play a protective role, Aβ transgenic worms CL4176 were transferred at the third larval (L3) stage (48 h after hatching) from MCCP to control dishes and the temperature was shifted to 25 °C to induce Aβ expression. As shown in Figure 3A, paralysis rates in worms that were transferred to control dishes from MCCP dishes were obviously decreased compared with worms maintained all the time on control dishes (although the protection was less than that of worms exposed to continual MCCP). 

This result showed that MCCP pretreatment to worms could still alleviate the toxicity of Aβ expression, even though there was no MCCP when Aβ began to express. We hypothesized that the protective effect of MCCP might be acted by induction of a previously identified stress response pathway. 

Oxidative stress has been greatly implicated along with the pathogenesis of age-related neurodegenerative diseases, such as AD [24]. It has also been reported that stress induced transcription factor (*skn-1*), heat shock factor protein 1 (*hsf-1*), and *daf-16* (a *FOXO*-family transcription factor) played important roles in regulating Aβ aggregation and then protected *C. elegans* from Aβ toxicity [21,25,26]. Besides, *sir-2.1* was related to stress resistance. Thus, we supposed whether ZTWE needed *sir-2.1*, *skn-1*, *hsf-1*, or *daf-16* to protect against Aβ toxicity in transgenic *C. elegans* CL4176. To confirm this, transcript abundances of *sir-2.1*, *skn-1*, *hsf-1* and *daf-16* were quantified. The results showed that transcript of *sir-2.1* was significantly upregulated, and it expressed by 1.4 fold compared to the control (Figure 3B). Because *sir-2.1* gene is necessary for the transcriptional activation of the *FOXO* transcription factor daf-16 target genes [27], we tested if the DAF-16 nuclear translocation could be promoted by MCCP, although the mRNA level of daf-16 had no change. Because of the link between oxidative stress and AD [24], we used a suite of transgenic GFP-reporter strains to investigate this possibility. We used the TJ356 (DAF-16::GFP) strain to determine whether MCCP was able to affect the cellular localization of DAF-16. DAF-16::GFP does not become localized in the nucleus under normal culturing conditions, but there is a rapid nuclear localization with heat treatment or oxidative stress with juglone [28]. Our results showed that treatment with MCCP accelerated the DAF-16 nuclear translocation (Figure 3C,D). In the nucleus, DAF-16 was known to activate transcription of a large number of genes that increase stress resistance and longevity, so we used the CF1553 (SOD-3::GFP) strain and the CF1588 (SOD-3::GFP; daf-2 and daf-16 double mutant) to test whether MCCP could increase the expression of SOD-3 by DAF-16. As the results showed, MCCP treatment could increase the expression of SOD-3 in CF1553 strain (Figure 3F) compared with the untreated worms (Figure 3E). While MCCP had no significant effect on SOD-3 expression in CF1588 strain (Figure 3G,H). The quantification of fluorescence intensity in *C. elegans* are shown in Figure 3I,J, which indicated that MCCP needed *daf-16* to increase SOD-3 expression. Combined with the previous results, it could be found that MCCP increased the expression of SOD-3 (downstream of DAF-16) by promoting nuclear translocation of DAF-16.

### 2.4. MCCP Increases Oxidative Stress Resistance in Transgenic C. elegans

**Figure 4:**
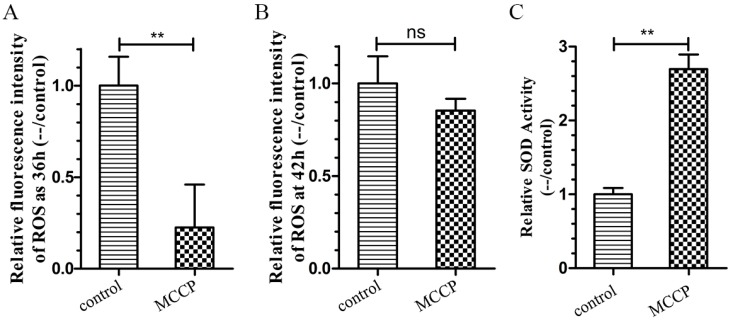
MCCP attenuated oxidative stress in *C. elegans*. (**A**,**B**) ROS were measured in MCCP treated and untreated CL4176 worms at 36 h and 42 h after temperature shifted to 25 °C. The ROS in worms was measured using 2′,7′-dichlorofluorescein. Results were expressed as DCF (2′,7′-dichlorofluorescein) fluorescence intensity relative to the untreated control. Data was obtained from three independent experiments, * *p* < 0.05, ** *p* < 0.01. (**C**) SOD activity was increased in CL4176 worms by treated with MCCP. Data was obtained from two independent experiments, ** *p* < 0.001.

The CL4176 worms treated with MCCP showed lower production of ROS in vivo measured through the H2DCF-DA (2′,7′-dichlorodihydrofluorescein diacetate) method (Figure 4A). The mean fluorescence intensity in transgenic worms with MCCP treatment at 36 h after temperature shifted to 25 °C was obviously reduced than the control worms. This trend was the same as 42 h after temperature shifted to 25 °C, although the difference was not significant (Figure 4B). Besides, as showed in Figure 4C, the activity of SOD-3 antioxidant enzymes was significantly increased in the MCCP treated worms compared with the control worms.

### 2.5. MCCP Has No Toxicity Effects to Wild Type C. elegans

**Figure 5:**
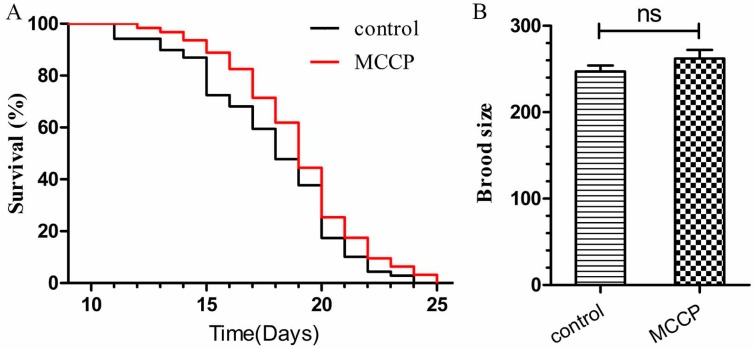
No toxicity effects of MCCP to *C. elegans* were found. (**A**) Wild-type N2 worms were treated with MCCP. The mean lifespan of worms treated with MCCP was 18.34 ± 2.82 days, which had a significant expansion compared with control worms (17.86 ± 3.17) (*p* < 0.05). The experiment was repeated three independent times. (**B**) The brood size of N2 worms treated with or without MCCP. MCCP treatment had no significant effect on brood size in *C. elegans*. Each experiment was repeated twice independent times.

To investigate if MCCP had any toxic effects to *C. elegans*, we employed the wild type *C. elegans* N2 for lifespan assay and brood size assay. As shown in Figure 5A, MCCP treatment not only had no toxicity, but also significantly prolonged the lifespan of worms. In addition, we measured the brood size of N2 in response to MCCP exposure and found no significant effects (Figure 5B). These results indicated that MCCP had no toxic effects to *C. elegans* and might be beneficial to worms.

## 3. Discussion

AD is a progressive neurodegenerative disease characterized by the accumulation of senile plaques in the brain forming mainly from Aβ, a peptide of 39–43 amino acids [29]. Aβ is produced by proteolytic cleavage of the transmembrane region of the amyloid precursor protein (APP) through sequential proteolytic processing by β- and γ-secretase [30]. Its soluble oligomeric forms accumulation could play a key role in the pathogenesis of AD [31]. Whether Aβ comes from the extracellular or intracellular site is still controversial, but it is increasingly recognized that intracellular Aβ is cytotoxic and an early, causative event in the development of AD, whereas extracellular Aβ seems to be the result of cell death and damages at a later stage [32].

Tea is being explored for the increase of lifespan and stress resistance [33] and the inhibition of amyloid-β oligomerization [7]. In the present study, we employed CL4176, a transgenic *C. elegans* strain induced expressing Aβ, as an AD model to investigate the protective effects of the water extract from a cultivar strain of the Pu’er tea, Zijuan (ZTWE) against Aβ toxicity in vivo. Excitingly, our results indicated that the supplementation of ZTWE substantially delayed the onset of amyloid-β-induced paralysis, and the effect was concentration dependent. We calculated the concentration of three main ingredients (+)-catechins, caffeine and procyanidins in ZTWE, and then detected the protective effects of (+)-catechins, caffeine, procyanidins and MCCP. The results showed that MCCP had a significant effect which was a little weaker than the effect of 0.4 mg/mL ZTWE but better than the effects of the single ones. MCCP played an important role in ZTWE. In addition, the effect of MCCP was better than that of the three individual components, which indicated that these three components played a synergistic role in *C. elegans* and enhanced the effect. We will elaborate on how these three components work synergistically in the following research. 

In order to explore how MCCP works, we first examined whether MCCP affects mRNA level of Aβ. We found that MCCP delayed the paralysis induced by Aβ-toxicity, while MCCP did not restrain *amy-1* transcript abundance in the worms, thereby suggesting that the biological effect of MCCP was acting at a post-transcriptional level. The thioflavin T staining results indicated that MCCP treated worms had fewer deposits than control worms. It suggested that MCCP-mediated protection against Aβ toxicity was, to some extent, through a reduction in the Aβ plaque deposition. 

AD pathology, specifically Aβ plaques, leads to oxidative stress which is associated with ROS accumulation that can further aggravate the pathological condition in AD patients [24,34]. We found that MCCP treatment decreased ROS content in the worms expressing Aβ (CL4176), which might be due to either the decreasing formation of toxic Aβ species or the increasing induction of the antioxidant system in worms, consequently reducing the negative effects of Aβ. Antioxidant therapies have been suggested as a potential method to alleviate pathology associated with AD [35], and MCCP may work in antioxidant therapies. 

**Figure 6:**
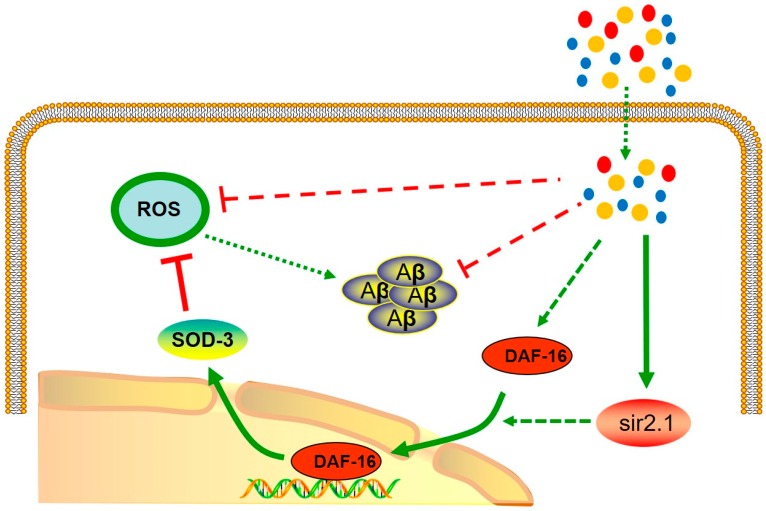
Pathways predicted to be involved in the anti-Alzheimer’s disease effect of MCCP. The green lines indicated the signal that up-regulated or activated the gene, and the red lines indicated the signal involved in the suppression. The dashed lines indicated the hypothetical pathway that remained to be elucidated. The yellow circle means (+)-catechins, the red circle means procyanidins and the blue circle means caffeine.

Previous studies reported that *daf-16*, *skn-1*, *hsf-1* and *sir-2.1* played pivotal roles in regulating longevity and ameliorating Aβ toxicity [21,23,25,36]. Thus, we wondered whether these regulators take part in the MCCP-mediated prevention against Aβ toxicity. To this end, we detected the transcriptional response of afore mentioned genes in CL4176 AD worms. Our results showed that *sir-2.1* was upregulated in CL4176 worms treated with MCCP as compared with controls without MCCP treatment. According to previous studies, SIR-2.1 activates DAF-16 [37]. In order to further study possible signaling pathways, we used the transgenic *C. elegans* TJ356 expressed DAF-16::GFP to detect the nuclear translocation of DAF-16 and we found that MCCP promoted the nuclear translocation of DAF-16. The transfer of DAF-16 from cytoplasm to nucleus is a necessary condition for DAF-16 to play its role in activating transcription and expression of downstream related factors [37]. We used the SOD::GFP worms to detect the expression of DAF-16 downstream SOD-3, and revealed that MCCP promoted the expression of SOD-3 in CF1553 (SOD-3::GFP) strain while ZTWE had no effects in CF1588 (SOD-3::GFP; *daf-2* and *daf-16* double mutant) strain. These results indicated that the expression of SOD-3 was promoted as a result of the nuclear translocation of DAF-16. Besides, the MCCP group promoted the nuclear translocation of DAF-16 and the expression of SOD after the worms were treated with juglone, which also verified that MCCP improved the ability of worms to resist oxidative stress by this pathway. The predictable signal pathway was shown in Figure 6.

## 4. Materials and Methods

### 4.1. Strains and Growth Conditions

*C. elegans* strain N2 (wild type), CL4176 dvIs27[pAF29(myo-3/A-Beta 1-42/let UTR) + pRF4(rol-6(su1006))], TJ356 zIs356 [*daf-16*p::*daf-16*a/b::GFP + rol-6], CF1553 muIs84 [(pAD76) sod-3p::GFP + rol-6] and CF1588:*daf-16* (mu86) I; *daf-2*(e1370) III; muIs84 [(pAD76) sod-3p::GFP + rol-6] were obtained from the *C. elegans* Genetic Center, CGC (University of Minnesota, Minneapolis, MN, USA). The transgenic worm strain CL4176 expressed muscle-specific Aβ_1–42_ [38] leading to a paralysis phenotype of the worm under non-permissive conditions. All *C. elegans* strains were maintained at 20 °C, except strain CL4176, which was maintained at 16 °C, on solid nematode growth medium (NGM), seeded with live *E. coli* (OP50) as a food source.

### 4.2. Preparation of Zijuan Pu’er Tea Extract

Zijuan Pu’er Tea powder was obtained from the Pu‘er Tianfu Biotechnology Development Co., Ltd. (Pu’er, China) which was available in shops or from the internet. Zijuan Pu’er tea powder (1 g) was extracted with boiling water (50 mL) for 20 min and the final volume was 50 mL. The storage concentration of the ZTWE was 20 mg/mL. 

The (+)-catechins and caffeine concentrations were determined by HPLC analysis which was conducted by using an LaChrom Elite L-2000 Liquid Chromatography system (Hitachi, Tokyo, Japan). The standards used were (+)-catechins (B21722, Yuanye, Shanghai, China) and caffeine (84677, Sigma, St. Louis, MO, USA). Isocratic elution was performed with a 3:7 ratio of solvent A (distilled water containing 0.1% formic acid) and solvent B (methanol), with a flow rate of 0.5 mL/min and duration for 20 min. The column was flushed with 100% B for 10 min and re-equilibrated for 5 min to the starting conditions for the next run. The UV detection acquisition wavelength was set at 280 nm and all determinations were performed at 25 °C. The injection volume was 20 µL. Before being used, the mobile phase was filtered through a 0.45 µm membrane filter (Millipore, Milford, MA, USA) and degassed under vacuum.

The procyanidin concentration was determined by the UV spectrophotometry. The standard used was procyanidin (CAS NO.4852-22-6, Biotopped, Beijing, China). Firstly, we configured different concentrations of procyanidin standard. Standard solution (1 mL), 5% hydrochloric acid/*n*-butanol solution (6 mL), and 2.0% ferric ammonium sulfate solution (0.2 mL) were mixed in a test tube, placed in a boiling water bath for 40 min, and then cooled in an ice bath for 15 min. Methanol (1 mL), 5% hydrochloric acid/*n*-butanol solution (6 mL), and 2.0% ferric ammonium sulfate solution (0.2 mL) were mixed in the other tube as a blank after boiling according to the above method. The absorbance was determined at 546 nm and a standard curve was drawn.

### 4.3. Life Span Assays

Lifespan assay was modified slightly according to the methods previously described [39,40]. Pregnant worms were allowed to lay eggs for 2–3 h to synchronize progeny on OP50 spread NGM plates with or without MCCP. When eggs reached young adult stages, approximately 100 worms were transferred onto new NGM dishes with or without MCCP and transferred every day. All lifespan assays were proceeded independently at 20 °C at least twice. Worms that were missing, attaching to walls or wormbag were not included in the life statistics but included in the analysis. 

### 4.4. Brood Size Assays

Total brood size measurement was modified slightly according to the methods previously described [39,40]. Each L4 stage hermaphrodite was transferred to a fresh NGM dish with or without MCCP at an interval of 24 h until the worms were no longer lay eggs. The brood size of each worm was the total number of hatched off-spring during the duration of the assay. Unhatched eggs were not counted as feasible offspring. Adult worms that were missing, adhering to the wall, dead or wormbag were excluded from the analysis. All brood size determinations were performed at least twice times independently at 20 °C.

### 4.5. Worm Paralysis Assays

*C. elegans* transgenic strain CL4176 was maintained at 16 °C on NGM dishes seeded with the *E. coli* strain OP50. For paralysis assay, synchronization of worms was performed on (+)-catechins, caffeine, procyanidins, MCCP or ZTWE treated and untreated dishes and were incubated at 16 °C (permissive temperature) for 48 h. After 48 h, the worms were transferred from 16 °C to 25 °C for cultivation [10]. Worms were scored at every two-hour interval until all the animals were paralyzed. Worms were scored as paralysis if they moved their heads only or failed to move their bodies by touching stimuluses with a platinum loop [4]. 

### 4.6. Staining of Aβ

36 h after temperature shifted from 16 °C to 25 °C, all the CL4176 transgenic worms with or without MCCP were washed from the dishes and transferred to microfuge tubes with 4% paraformaldehyde/M9 buffer, pH 7.4, for 24 h at 4 °C. Fixed animals were permeabilized in 5% fresh β-mercaptoethanol, 1% Triton X-100, 125 mM Tris pH 7.4, in a 37 °C incubator for 24 h. After washing 2 times with M9 buffer, the samples were stained with 0.125% thioflavin T (Sigma) in 50% ethanol for 2 min and destained with sequential ethanol washes (50%, 75%, 90%, 75%, and 50% *v*/*v*), and each for 2 min. The worms were washed with M9 buffer containing 1% Triton X-100 and then the animals were finally transferred to slides using a drop of M9 buffer and fluorescence images were acquired by a fluorescence microscope (AMG EVOS FL, Westover Scientific, Seattle, WA, USA) [7]. 

### 4.7. DAF-16 Localization via Fluorescence Microscopy

The TJ356 strain was used to examine the intracellular distribution of DAF-16 in the living worms [41]. In this strain, the *daf-16* gene was fused to the gene coding for green fluorescent protein (GFP). TJ356 worms were cultivated with or without MCCP for 4 days, and then were adopted the effect of oxidative stress by 200 μM juglone for 2 h. DAF-16 localization was examined in approximately 10 animals per treatment that were mounted in a drop of 10 mM sodium azide. Fluorescence images were taken at a constant exposure time by an IX71 fluorescence microscope (OLYMPUS, Tokyo, Japan).

### 4.8. SOD-3 Expression via Fluorescence Microscopy

The CF1553 strain and CF1588 strain were used to examine the expression of SOD-3 in the living worms. In these strains, the gene coding for green fluorescent protein (GFP) was fused to the *sod-3* gene while the CF1588 strain was *daf-2* and *daf-16* double-mutant. CF1553 and CF1588 worms were cultivated with or without MCCP for 4 days, and then they were adopted the effect of oxidative stress by 200 μM juglone for 2 h. SOD-3 expression was examined in approximately 10 animals per treatment that were mounted in a drop of 10 mM sodium azide. Fluorescence images were taken at constant exposure times by a fluorescence microscope (OLYMPUS IX71, Tokyo, Japan).

### 4.9. Measurement of ROS and SOD Activity in C. elegans

Intracellular ROS were measured in transgenic *C. elegans* strain CL4176, using the 2′,7′-dichlorofluorescein diacetate (H2DCF-DA) method [4,42,43]. Briefly, synchronization of worms was performed on fresh NGM dishes with or without MCCP and were incubated at 16 °C (permissive temperature) for 48 h. To initiate amyloid-induced progressive paralysis, the worms were transferred from 16 °C to 25 °C for cultivation. 200 worms of each group were collected at 36 h and 42 h after the temperature shifted to 25 °C using 500 μL M9 buffer, washed twice with M9 buffer to remove *E. coli* (OP50) bacteria and then transferred into tubes with 500 μL M9 buffer containing Tween 20 (0.01%) and H2DCF-DA (50 μM). After incubated for 2 h, the worms were washed by M9 buffer for two times to remove unbounded H2DCF-DA. Then the worms were transferred to 96-well plates and each well contained 200 μL M9 buffer and 20 worms. Each group had five parallel wells. Then the PBS-*C. elegans* mixture was placed at the plate for fluorescence detection by a fluorescence microplate reader (Bio-Tek Instruments, Winooski, VT, USA).

SOD Activity in *C. elegans* detected by the autoxidation of pyrogallol method. Briefly, synchronization of worms was performed on fresh NGM dishes with or without MCCP and were incubated at 16 °C (permissive temperature) for 48 h. To initiate amyloid-induced progressive paralysis, the worms were transferred from 16 °C to 25 °C for cultivation. 100 worms were collected in every dish at 36h after the temperature shifted to 25 °C using 500 μL of M9 buffer and they were broken by ultrasound. First, the rate of pyrogallol autoxidation was detected using UV-vis spectrophotometer (PERSEE TU-1900, PERSEE General Instrument Co, Ltd, Beijing, China). Next, worms crushing liquids of different groups were added in pyrogallol solution, and the oxidation rates of different groups were detected using UV-vis spectrophotometer. Finally, the SOD activities were calculated according to the above values.

### 4.10. Expression Analysis by Quantitative Real Time RCR

Quantitative real-time RCR was performed as previously described with minor modifications [4]. Quantitative real-time PCR was proceeded with CL4176 worms treated with or without MCCP in order to link their phenotypic and biochemical responses with the molecular response, under conditions leading to expression of Aβ protein species in the worms. For this, the transcriptional response of amyloid-β transgene (*amy-1*), stress induced transcription factor (*skn-1*), heat shock factor protein 1 (*hsf-1*), *daf-16*, and *sir-2.1* were detected. Eggs were transferred to control and MCCP treatment dishes and incubated for 48h at permissive temperature (16 °C) and then shifted to 25 °C to induce *amy-1* expression. Temperature was up-shifted to 25 °C for 36 h, the CL4176 worms were collected and washed with M9 buffer for two times, and then transferred directly into Trizol Reagent and flash frozen at −80 °C. The worms were broken by multigelation. Total RNA was extracted with TRIzol reagent following standard protocol and cDNA was synthesized with a TransScript One-Step gDNA Removal and cDNA Synthesis SuperMix Kit (Transgen Biology, Beijing, China). The RT-PCR primers were as follows and the gene *act-1* was used as the internal control.

*amy-1*:forward, 5′-CCGACATGACTCAGGATATGAAGT-3′,
reverse, 5′-CACCATGAGTCCAATGATTGCA-3′;*sir-2.1*:forward, 5′-AGAACGCGCATTTCGCCATATTAAG-3′,
reverse, 5′-ATACTGACACTCCAGCGCCAG-3′;*skn-1*:forward, 5′-AGTGTCGGCGTTCCAGATTTC-3′,
reverse, 5′-GTCGACGAACTTGCGAATCA-3′;*hsf-1*:forward, 5′-TTGACGACGACAAGCTTCCAGT-3′,
reverse, 5′-AAAGCTTGCACCAGAATCATCCC-3′;*daf-16*:forward, 5′-TTTCCGTCCCCGAACTCAA-3′,
reverse, 5′-ATTCGCCAACCCATGATGG-3′;*act-1*:forward, 5′-CCAGGAATTGCTGATCGTATGCAGAA-3′,
reverse, 5′-TGGAGAGGGAAGCGAGGATAGA-3′.

### 4.11. Statistical Analysis

Statistical analysis was performed using Prism 5 software (GraphPad Software, Inc., La Jolla, CA, USA). The paralysis and lifespan of nematodes cultured in the absence or presence of MCCP was compared between groups using the two-tailed, unpaired Student’s *t*-test. Data other than paralysis and lifespan were analyzed using one-way analysis of variance (ANOVA). Results were expressed as the mean ± standard deviation of three independent experiments. *p*-Values < 0.05 was taken as statistically significant (0.01 ≤ * *p* < 0.05, 0.001 ≤ ** *p* < 0.01, *** *p* < 0.001).

## 5. Conclusions

Our findings elucidated some of the molecular mechanisms by which MCCP delayed Aβ toxicity in *C. elegans* and illuminated the beneficial effects of MCCP on AD prevention. MCCP reduced deposition of Aβ, increased antioxidant activity, and might activate *daf-16* pathway in the transgenic *C. elegans* model that enhanced the ability of oxidative stress resistance in *C. elegans*. Although health effects of Zijuan Pu’er tea have been studied for many years, our research is the first to study the properties of ZTWE and the mixture of its ingredients on protection against Aβ toxicity and the first to systematically analyze the genetic mechanism for MCCP-mediated anti-AD effects. The results further also advise to investigate if these health benefits are obtained with dietary Zijuan Pu’er tea in higher order organisms.

## Figures and Tables

**Table 1:** Standard curves of (+)-catechins, caffeine and procyanidins.

	Standard Curve	R^2^
(+)-catechins	Y = (1.53376 × 10^7^)X + 47476.3	0.9997
caffeine	Y = (7.26906 × 10^7^)X + 245960	0.9995
procyanidins	Y = 0.242X + 0.0041	0.9983
